# {μ-6,6′-Dimeth­oxy-2,2′-[1,2-phenyl­ene­bis(nitrilo­methyl­idyne)]diphenolato}methano­lcopper(II)sodium(I)

**DOI:** 10.1107/S1600536808008453

**Published:** 2008-04-04

**Authors:** Jiang Bian

**Affiliations:** a South China Sea Institute of Oceanology, Chinese Academy of Sciences, Guangzhou 510301, People’s Republic of China, and Institute of Microbiology, Chinese Academy of Sciences, Beijing 100101, People’s Republic of China, and Graduate University of Chinese Academy of Sciences, Chinese Academy of Sciences, Beijing 100049, People’s Republic of China

## Abstract

In the title complex, [NaCu(C_22_H_18_N_2_O_4_)Cl(CH_3_OH)], the Cu atom lies nearly in the plane defined by the N_2_O_2_ core of donor atoms, the out-of-plane distance being 0.001 (2) Å. The anion provides a planar cavity of four O atoms which accommodates a sodium cation. The coordination geometry around sodium is completed by the methanol O atom and a chloride ion. The four O atoms define a coordination plane containing the sodium cation [maximum displacement from the mean plane through the five atoms = 0.152 (3) Å for Na]. The crystal structure is stabilized by inter­molecular C—H⋯Cl and O—H⋯Cl hydrogen bonds, which link the mol­ecules into dimers. The crystal packing is further stabilized by weak π–π stacking inter­actions [centroid–centroid distances of 3.442 (4), 3.482 (3), 3.350 (2), 3.531 (4) 3.575 (2) and 3.604 (2) Å].

## Related literature

For related literature, see: Molina *et al.* (1998 ▶); Lo *et al.* (2004 ▶, 2006 ▶).
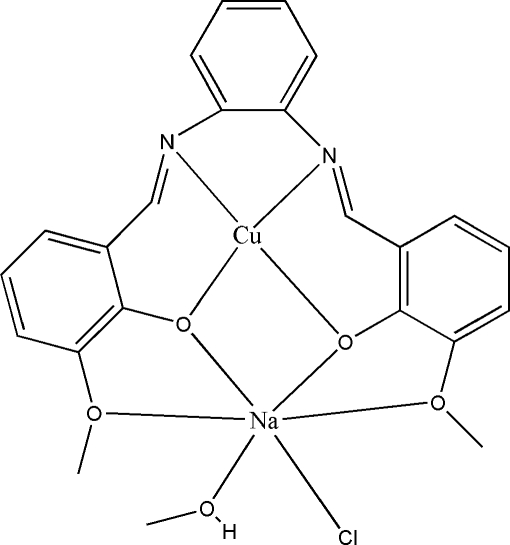


## Experimental

### 

#### Crystal data

[NaCu(C_22_H_18_N_2_O_4_)Cl(CH_4_O)]
*M* *_r_* = 528.41
Monoclinic, 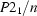

*a* = 11.7986 (2) Å
*b* = 7.9657 (2) Å
*c* = 23.8291 (3) Å
β = 93.283 (2)°
*V* = 2235.88 (7) Å^3^
*Z* = 4
Mo *K*α radiation
μ = 1.16 mm^−1^
*T* = 295 (2) K
0.22 × 0.18 × 0.12 mm

#### Data collection

Bruker APEX CCD area-detector diffractometer
Absorption correction: multi-scan (*SADABS*; Sheldrick, 2004 ▶) *T*_min_ = 0.785, *T*_max_ = 0.874
20679 measured reflections
4620 independent reflections
3958 reflections with *I* > 2σ(*I*)
*R*_int_ = 0.018

#### Refinement

*R*[*F* ^2^ > 2σ(*F* ^2^)] = 0.028
*wR*(*F* ^2^) = 0.083
*S* = 1.02
4620 reflections
302 parameters
1 restraint
H atoms treated by a mixture of independent and constrained refinement
Δρ_max_ = 0.39 e Å^−3^
Δρ_min_ = −0.24 e Å^−3^

### 

Data collection: *SMART* (Bruker, 2001 ▶); cell refinement: *SAINT* (Bruker, 2001 ▶); data reduction: *SAINT*; program(s) used to solve structure: *SHELXTL* (Sheldrick, 2008 ▶); program(s) used to refine structure: *SHELXTL*; molecular graphics: *SHELXTL*; software used to prepare material for publication: *SHELXTL* and local programs.

## Supplementary Material

Crystal structure: contains datablocks I, global. DOI: 10.1107/S1600536808008453/hg2383sup1.cif

Structure factors: contains datablocks I. DOI: 10.1107/S1600536808008453/hg2383Isup2.hkl

Additional supplementary materials:  crystallographic information; 3D view; checkCIF report

## Figures and Tables

**Table 1:** Hydrogen-bond geometry (Å, °)

*D*—H⋯*A*	*D*—H	H⋯*A*	*D*⋯*A*	*D*—H⋯*A*
O5—H5*A*⋯Cl1^i^	0.86 (2)	2.34 (2)	3.193 (2)	172 (4)
C10—H10⋯Cl1^ii^	0.93	2.82	3.729 (2)	165

Symmetry codes: (i) 
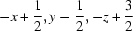
; (ii) 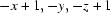.

**Fig. 1.**
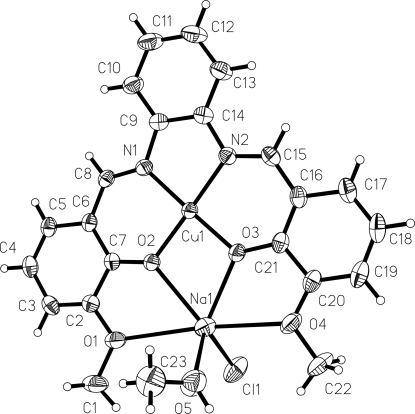
View of the title compound (I), with displacement ellipsoids drawn at the 40% probability level.

**Fig. 2.**
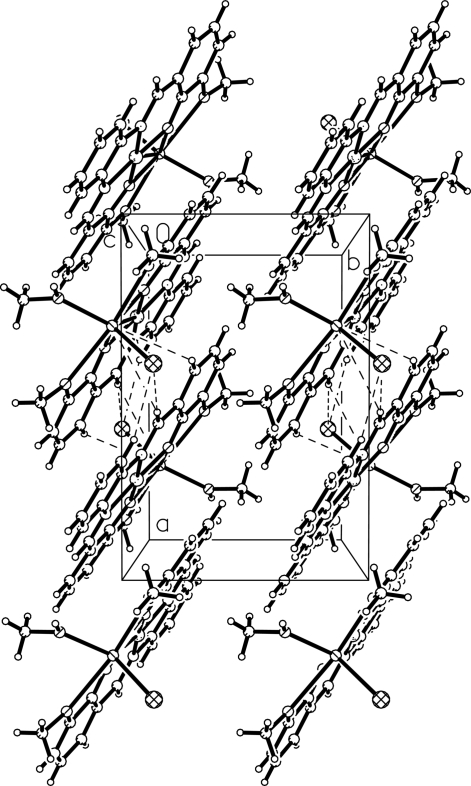
Packing diagram of structure of (I), view along the c axis. Hydrogen bonds are shown as dashed lines.

## supplementary crystallographic information

### Comment

It is known that phenylene bridged Schiff base ligand *N*,*N*'-1,2-Phenylene-bis(3-methoxysalicylideneiminato) as N– or O-donors exhibit excellent coordination capability to form supramolecular frameworks. Self-assembly by H-bonding, π-π stacking, and van der Waals interactions is also an important process in the formation of noncovalent supramolecular frameworks (Lo *et al.*, 2006; Molina *et al.*, 1998). The title compound, (I), was prepared by employing *N*,*N*'-1,2-Phenylene-bis(3-methoxysalicylideneiminato) as ligand. Here we present its crystal structure.

In the title complex,(I), the copper atoms lie nearly in the plane defined by the N_2_O_2_ core of donor atoms, the out-of-plane distances being 0.001 (2)Å. The anion provides a planar cavity of four oxygen atoms which accommodates a sodium cation. The coordination geometry around sodium is completed by the oxygen atom from methanol molecule and chloride ion. The four oxygen atoms [O1,O2,O3,O4] define a c0ordination plane containing the sodium cation (maximum displacement from the mean plane through the five atoms: 0.152 (3) Å for Na). The crystal structure is stabilized by intermolecular C—H···Cl hydrogen bonds, which link the molecules into dimers. The packing is also stabilized by six intermolecular π-π stacking interactions, with relatively short distance *Cg*1···*Cg*4^i^ 3.575 (2) Å, *Cg*1···*Cg*5^ii^ 3.482 (3) Å, *Cg*2···*Cg*2^i^ 3.604 (2) Å, *Cg*2···*Cg*4^i^ 3.350 (2) Å, *Cg*3···*Cg*3^ii^ 3.442 (4) Å, *Cg*3···*Cg*5^ii^ 3.531 (4) Å, where *Cg*1, *Cg*2, *Cg*3, *Cg*4 and *Cg*5 are centroids of Cu1/N1/N2/C9/C14, Cu1/O2/N1/C6—C8, Cu1/O3/N2/C15/C16/C21, C2—C7 and C16—C21 rings, respectively [symmetry code: (i) 2 - *x*,1 - *y*,-*z*, (ii) 1 - *x*,1 - *y*,-*z*].

### Experimental

The phenylene bridged Schiff base ligand *N*,*N*'-1,2-Phenylene-bis(3-methoxysalicylideneiminato) was prepared in excellent yield (92%) according to the literature method (Lo *et al.*, 2004) *via* the condensation of 1,2-diaminobenzene with *o*-vanillin and 5-(4'-methylphenyl)-3-methoxysalicylaldehyde, respectively, in a 2:1 mole ratio. The ^1^H NMR spectrum of Schiff base ligand in CDCl3 showed a singlet at δ 8.60 for the imino protons and a broad singlet at δ 13.23 for the hydroxyl protons, respectively.

To a solution of the *N*,*N*'-1,2-Phenylene-bis(3-methoxysalicylideneiminato (0.38 g, 1 mmol) in methanol (10 ml), 0.134 g (1 mmol) CuCl_2_, 0.06 g (1 mmol) NaCl powder was slowly added. After stirring for four hours, the solution was filtered to remove the precipitate and placed in a desiccator filled with methanol. Brown crystals were obtained about one week later. Elemental analysis [found (calculated)] for C_23_H_22_N_2_O_5_ClCuNa: C 52.20 (52.28), H 4.23 (4.20), N 5.20% (5.30%). IR /m C=N 1642 cm^-1^.

### Refinement

All H atoms were found on difference maps. The hydroxy proton  atoms were refined freely, giving an O–H bond distance of 0.86 Å. The remaining atoms were placed in calculated positions, with C—H = 0.93 or 0.96 Å, and included in the final cycles of refinement using a riding model, with *U*_iso_(H) = 1.2 times *U*_eq_(C) (1.5 *U*_eq_ for methyl).

### Crystal data

**Table:**

[NaCu(C_22_H_18_N_2_O_4_)Cl(CH_4_O)]	*F*_000_ = 1084
*M**_r_* = 528.41	*D*_x_ = 1.570 Mg m^−^^3^
Monoclinic, *P*2_1_/*n*	Mo *K*α radiation λ = 0.71073 Å
Hall symbol: -P 2yn	Cell parameters from 3689 reflections
*a* = 11.7986 (2) Å	θ = 2.2–26.6º
*b* = 7.9657 (2) Å	µ = 1.16 mm^−^^1^
*c* = 23.8291 (3) Å	*T* = 295 (2) K
β = 93.283 (2)º	Block, brown
*V* = 2235.88 (7) Å^3^	0.22 × 0.18 × 0.12 mm
*Z* = 4	

### Data collection

**Table:**

Bruker APEX CCD area-detector diffractometer	4620 independent reflections
Radiation source: fine-focus sealed tube	3958 reflections with *I* > 2σ(*I*)
Monochromator: graphite	*R*_int_ = 0.018
Detector resolution: 0 pixels mm^-1^	θ_max_ = 26.5º
*T* = 295(2) K	θ_min_ = 1.7º
φ and ω scans	*h* = −14→14
Absorption correction: multi-scan(SADABS; Sheldrick, 2004)	*k* = −10→10
*T*_min_ = 0.785, *T*_max_ = 0.874	*l* = −27→29
20679 measured reflections	

### Refinement

**Table:**

Refinement on *F*^2^	Secondary atom site location: difference Fourier map
Least-squares matrix: full	Hydrogen site location: inferred from neighbouring sites
*R*[*F*^2^ > 2σ(*F*^2^)] = 0.028	H atoms treated by a mixture of independent and constrained refinement
*wR*(*F*^2^) = 0.083	*w* = 1/[σ^2^(*F*_o_^2^) + (0.0451*P*)^2^ + 1.0557*P*] where *P* = (*F*_o_^2^ + 2*F*_c_^2^)/3
*S* = 1.02	(Δ/σ)_max_ = 0.001
4620 reflections	Δρ_max_ = 0.39 e Å^−^^3^
302 parameters	Δρ_min_ = −0.24 e Å^−^^3^
1 restraint	Extinction correction: none
Primary atom site location: structure-invariant direct methods	

### Special details

**Table:**

Geometry. All e.s.d.'s (except the e.s.d. in the dihedral angle between two l.s. planes) are estimated using the full covariance matrix. The cell e.s.d.'s are taken into account individually in the estimation of e.s.d.'s in distances, angles and torsion angles; correlations between e.s.d.'s in cell parameters are only used when they are defined by crystal symmetry. An approximate (isotropic) treatment of cell e.s.d.'s is used for estimating e.s.d.'s involving l.s. planes.
Refinement. Refinement of *F*^2^ against ALL reflections. The weighted *R*-factor *wR* and goodness of fit *S* are based on *F*^2^, conventional *R*-factors *R* are based on *F*, with *F* set to zero for negative *F*^2^. The threshold expression of *F*^2^ > σ(*F*^2^) is used only for calculating *R*-factors(gt) *etc*. and is not relevant to the choice of reflections for refinement. *R*-factors based on *F*^2^ are statistically about twice as large as those based on *F*, and *R*- factors based on ALL data will be even larger.

### Fractional atomic coordinates and isotropic or equivalent isotropic displacement parameters (Å^2^)

**Table:**

	*x*	*y*	*z*	*U*_iso_*/*U*_eq_	
Cu1	0.245877 (17)	0.00018 (3)	0.488480 (9)	0.03216 (8)	
Na1	0.28232 (7)	−0.09373 (11)	0.62911 (3)	0.0476 (2)	
N1	0.32603 (13)	−0.03020 (18)	0.42070 (6)	0.0325 (3)	
N2	0.14259 (12)	0.13666 (19)	0.44095 (6)	0.0343 (3)	
O1	0.45834 (12)	−0.3066 (2)	0.61004 (6)	0.0500 (4)	
O2	0.34391 (10)	−0.14186 (16)	0.53338 (5)	0.0364 (3)	
O3	0.16904 (11)	0.04016 (17)	0.55486 (5)	0.0387 (3)	
O4	0.10358 (13)	0.0706 (2)	0.65442 (6)	0.0577 (4)	
O5	0.20263 (19)	−0.3239 (2)	0.67333 (9)	0.0760 (5)	
Cl1	0.39839 (5)	0.09785 (10)	0.70798 (3)	0.0707 (2)	
C1	0.5137 (2)	−0.4081 (4)	0.65277 (10)	0.0664 (7)	
H1A	0.4732	−0.4008	0.6865	0.100*	
H1B	0.5900	−0.3690	0.6602	0.100*	
H1C	0.5150	−0.5227	0.6404	0.100*	
C2	0.50487 (15)	−0.3024 (2)	0.55889 (8)	0.0358 (4)	
C3	0.60488 (16)	−0.3766 (2)	0.54631 (9)	0.0410 (4)	
H3	0.6460	−0.4385	0.5736	0.049*	
C4	0.64579 (16)	−0.3597 (2)	0.49238 (9)	0.0427 (4)	
H4	0.7142	−0.4096	0.4842	0.051*	
C5	0.58578 (15)	−0.2708 (2)	0.45224 (9)	0.0382 (4)	
H5	0.6142	−0.2593	0.4168	0.046*	
C6	0.48040 (14)	−0.1950 (2)	0.46330 (8)	0.0324 (4)	
C7	0.43811 (14)	−0.2087 (2)	0.51781 (7)	0.0308 (4)	
C8	0.42240 (14)	−0.1073 (2)	0.41826 (7)	0.0334 (4)	
H8	0.4566	−0.1051	0.3841	0.040*	
C9	0.27193 (15)	0.0517 (2)	0.37354 (8)	0.0360 (4)	
C10	0.30926 (19)	0.0474 (3)	0.31902 (9)	0.0488 (5)	
H10	0.3754	−0.0099	0.3116	0.059*	
C11	0.2474 (2)	0.1286 (3)	0.27618 (9)	0.0604 (6)	
H11	0.2718	0.1248	0.2398	0.072*	
C12	0.1496 (2)	0.2153 (3)	0.28688 (10)	0.0603 (6)	
H12	0.1087	0.2693	0.2576	0.072*	
C13	0.11237 (18)	0.2224 (3)	0.34051 (9)	0.0506 (5)	
H13	0.0468	0.2818	0.3475	0.061*	
C14	0.17298 (15)	0.1405 (2)	0.38442 (8)	0.0371 (4)	
C15	0.05522 (15)	0.2153 (2)	0.45833 (8)	0.0395 (4)	
H15	0.0102	0.2733	0.4315	0.047*	
C16	0.02163 (15)	0.2213 (2)	0.51487 (9)	0.0391 (4)	
C17	−0.07586 (17)	0.3182 (3)	0.52576 (11)	0.0521 (5)	
H17	−0.1154	0.3727	0.4962	0.063*	
C18	−0.11214 (19)	0.3325 (3)	0.57844 (11)	0.0603 (6)	
H18	−0.1764	0.3958	0.5847	0.072*	
C19	−0.05333 (18)	0.2524 (3)	0.62352 (10)	0.0545 (6)	
H19	−0.0778	0.2646	0.6597	0.065*	
C20	0.04028 (16)	0.1560 (3)	0.61461 (9)	0.0429 (4)	
C21	0.08043 (14)	0.1367 (2)	0.55956 (8)	0.0356 (4)	
C22	0.0796 (2)	0.0956 (4)	0.71220 (9)	0.0702 (7)	
H22A	0.1302	0.0282	0.7358	0.105*	
H22B	0.0026	0.0635	0.7177	0.105*	
H22C	0.0899	0.2118	0.7218	0.105*	
C23	0.2056 (3)	−0.4837 (4)	0.64857 (15)	0.0820 (9)	
H23A	0.1700	−0.5636	0.6721	0.123*	
H23B	0.2831	−0.5160	0.6444	0.123*	
H23C	0.1658	−0.4807	0.6123	0.123*	
H5A	0.172 (4)	−0.335 (6)	0.7048 (10)	0.161 (19)*	

### Atomic displacement parameters (Å^2^)

**Table:**

	*U*^11^	*U*^22^	*U*^33^	*U*^12^	*U*^13^	*U*^23^
Cu1	0.02835 (13)	0.03863 (14)	0.02980 (13)	0.00368 (8)	0.00441 (8)	0.00103 (8)
Na1	0.0472 (4)	0.0560 (5)	0.0399 (4)	−0.0035 (4)	0.0041 (3)	0.0006 (4)
N1	0.0334 (8)	0.0340 (8)	0.0302 (8)	−0.0024 (6)	0.0040 (6)	0.0005 (6)
N2	0.0304 (7)	0.0360 (8)	0.0364 (8)	−0.0001 (6)	0.0008 (6)	0.0012 (6)
O1	0.0527 (8)	0.0636 (9)	0.0337 (7)	0.0093 (7)	0.0017 (6)	0.0075 (7)
O2	0.0328 (6)	0.0442 (7)	0.0327 (6)	0.0075 (5)	0.0062 (5)	0.0032 (5)
O3	0.0327 (6)	0.0502 (7)	0.0337 (7)	0.0075 (6)	0.0069 (5)	−0.0006 (6)
O4	0.0513 (9)	0.0869 (12)	0.0361 (7)	0.0088 (8)	0.0126 (6)	−0.0058 (8)
O5	0.0989 (15)	0.0591 (11)	0.0731 (13)	−0.0056 (10)	0.0331 (11)	0.0029 (10)
Cl1	0.0488 (3)	0.1063 (5)	0.0580 (3)	−0.0131 (3)	0.0121 (3)	−0.0248 (4)
C1	0.0759 (17)	0.0821 (18)	0.0401 (12)	0.0095 (14)	−0.0066 (11)	0.0158 (12)
C2	0.0365 (9)	0.0344 (9)	0.0361 (9)	−0.0024 (7)	−0.0001 (7)	−0.0008 (7)
C3	0.0361 (9)	0.0351 (9)	0.0506 (11)	0.0030 (8)	−0.0073 (8)	0.0010 (8)
C4	0.0317 (9)	0.0386 (10)	0.0580 (12)	0.0041 (8)	0.0054 (8)	−0.0057 (9)
C5	0.0340 (9)	0.0352 (9)	0.0463 (11)	−0.0007 (7)	0.0104 (8)	−0.0040 (8)
C6	0.0309 (8)	0.0287 (8)	0.0381 (9)	−0.0024 (7)	0.0059 (7)	−0.0036 (7)
C7	0.0292 (8)	0.0284 (8)	0.0349 (9)	−0.0024 (7)	0.0015 (7)	−0.0024 (7)
C8	0.0343 (9)	0.0343 (9)	0.0323 (9)	−0.0037 (7)	0.0081 (7)	−0.0028 (7)
C9	0.0361 (9)	0.0382 (9)	0.0336 (9)	−0.0056 (8)	0.0000 (7)	0.0024 (8)
C10	0.0486 (12)	0.0625 (13)	0.0356 (10)	0.0021 (10)	0.0063 (9)	0.0027 (10)
C11	0.0619 (14)	0.0848 (17)	0.0345 (11)	−0.0027 (13)	0.0040 (10)	0.0124 (11)
C12	0.0591 (14)	0.0755 (16)	0.0450 (12)	0.0012 (12)	−0.0069 (10)	0.0210 (12)
C13	0.0440 (11)	0.0599 (13)	0.0472 (12)	0.0038 (10)	−0.0029 (9)	0.0123 (10)
C14	0.0352 (9)	0.0388 (10)	0.0372 (9)	−0.0050 (7)	−0.0001 (7)	0.0038 (8)
C15	0.0322 (9)	0.0384 (10)	0.0474 (11)	0.0013 (7)	−0.0018 (8)	0.0050 (8)
C16	0.0293 (9)	0.0369 (9)	0.0516 (11)	−0.0008 (7)	0.0056 (8)	−0.0034 (9)
C17	0.0375 (10)	0.0491 (12)	0.0702 (15)	0.0103 (9)	0.0072 (10)	0.0008 (11)
C18	0.0408 (11)	0.0566 (13)	0.0852 (18)	0.0111 (10)	0.0194 (12)	−0.0115 (13)
C19	0.0432 (11)	0.0598 (13)	0.0627 (14)	−0.0020 (10)	0.0216 (10)	−0.0168 (12)
C20	0.0348 (9)	0.0482 (11)	0.0466 (11)	−0.0054 (8)	0.0102 (8)	−0.0108 (9)
C21	0.0270 (8)	0.0378 (9)	0.0428 (10)	−0.0051 (7)	0.0074 (7)	−0.0071 (8)
C22	0.0670 (15)	0.108 (2)	0.0375 (12)	−0.0051 (15)	0.0183 (11)	−0.0125 (13)
C23	0.083 (2)	0.0695 (19)	0.093 (2)	−0.0177 (15)	0.0020 (18)	−0.0110 (15)

### Geometric parameters (Å, °)

**Table:**

Cu1—O3	1.8947 (13)	C5—C6	1.420 (2)
Cu1—O2	1.9029 (12)	C5—H5	0.9300
Cu1—N1	1.9331 (15)	C6—C7	1.422 (2)
Cu1—N2	1.9472 (15)	C6—C8	1.423 (3)
Cu1—Na1	3.4363 (8)	C8—H8	0.9300
Na1—O5	2.339 (2)	C9—C10	1.396 (3)
Na1—O3	2.4047 (15)	C9—C14	1.402 (3)
Na1—O2	2.4633 (14)	C10—C11	1.381 (3)
Na1—O4	2.5828 (17)	C10—H10	0.9300
Na1—Cl1	2.7279 (10)	C11—C12	1.380 (4)
Na1—O1	2.7392 (17)	C11—H11	0.9300
N1—C8	1.297 (2)	C12—C13	1.376 (3)
N1—C9	1.419 (2)	C12—H12	0.9300
N2—C15	1.295 (2)	C13—C14	1.395 (3)
N2—C14	1.414 (2)	C13—H13	0.9300
O1—C2	1.365 (2)	C15—C16	1.427 (3)
O1—C1	1.429 (3)	C15—H15	0.9300
O2—C7	1.305 (2)	C16—C21	1.409 (3)
O3—C21	1.308 (2)	C16—C17	1.421 (3)
O4—C20	1.356 (3)	C17—C18	1.354 (3)
O4—C22	1.435 (2)	C17—H17	0.9300
O5—C23	1.404 (3)	C18—C19	1.399 (4)
O5—H5A	0.86 (3)	C18—H18	0.9300
C1—H1A	0.9600	C19—C20	1.372 (3)
C1—H1B	0.9600	C19—H19	0.9300
C1—H1C	0.9600	C20—C21	1.428 (3)
C2—C3	1.368 (3)	C22—H22A	0.9600
C2—C7	1.431 (3)	C22—H22B	0.9600
C3—C4	1.405 (3)	C22—H22C	0.9600
C3—H3	0.9300	C23—H23A	0.9600
C4—C5	1.356 (3)	C23—H23B	0.9600
C4—H4	0.9300	C23—H23C	0.9600
			
O3—Cu1—O2	86.27 (5)	C5—C4—H4	119.9
O3—Cu1—N1	177.46 (6)	C3—C4—H4	119.9
O2—Cu1—N1	94.86 (6)	C4—C5—C6	121.22 (18)
O3—Cu1—N2	94.49 (6)	C4—C5—H5	119.4
O2—Cu1—N2	177.46 (6)	C6—C5—H5	119.4
N1—Cu1—N2	84.47 (6)	C5—C6—C7	119.57 (16)
O3—Cu1—Na1	42.40 (4)	C5—C6—C8	117.21 (16)
O2—Cu1—Na1	44.29 (4)	C7—C6—C8	123.23 (15)
N1—Cu1—Na1	138.39 (5)	O2—C7—C6	125.27 (16)
N2—Cu1—Na1	136.71 (5)	O2—C7—C2	117.55 (16)
O5—Na1—O3	117.28 (7)	C6—C7—C2	117.18 (15)
O5—Na1—O2	116.28 (7)	N1—C8—C6	125.73 (16)
O3—Na1—O2	64.46 (4)	N1—C8—H8	117.1
O5—Na1—O4	86.42 (7)	C6—C8—H8	117.1
O3—Na1—O4	61.32 (5)	C10—C9—C14	119.68 (18)
O2—Na1—O4	125.68 (5)	C10—C9—N1	125.01 (18)
O5—Na1—Cl1	109.09 (6)	C14—C9—N1	115.30 (16)
O3—Na1—Cl1	119.65 (5)	C11—C10—C9	119.6 (2)
O2—Na1—Cl1	124.19 (4)	C11—C10—H10	120.2
O4—Na1—Cl1	86.56 (5)	C9—C10—H10	120.2
O5—Na1—O1	85.27 (7)	C12—C11—C10	120.6 (2)
O3—Na1—O1	123.18 (5)	C12—C11—H11	119.7
O2—Na1—O1	58.89 (4)	C10—C11—H11	119.7
O4—Na1—O1	171.68 (6)	C13—C12—C11	120.4 (2)
Cl1—Na1—O1	96.03 (4)	C13—C12—H12	119.8
O5—Na1—Cu1	125.38 (6)	C11—C12—H12	119.8
O3—Na1—Cu1	32.09 (3)	C12—C13—C14	120.0 (2)
O2—Na1—Cu1	32.65 (3)	C12—C13—H13	120.0
O4—Na1—Cu1	93.39 (4)	C14—C13—H13	120.0
Cl1—Na1—Cu1	125.44 (3)	C13—C14—C9	119.59 (18)
O1—Na1—Cu1	91.53 (4)	C13—C14—N2	125.29 (18)
C8—N1—C9	122.57 (16)	C9—C14—N2	115.12 (16)
C8—N1—Cu1	124.68 (13)	N2—C15—C16	125.98 (18)
C9—N1—Cu1	112.62 (12)	N2—C15—H15	117.0
C15—N2—C14	122.97 (16)	C16—C15—H15	117.0
C15—N2—Cu1	124.58 (13)	C21—C16—C17	119.37 (19)
C14—N2—Cu1	112.45 (11)	C21—C16—C15	123.13 (16)
C2—O1—C1	117.35 (17)	C17—C16—C15	117.50 (19)
C2—O1—Na1	118.69 (11)	C18—C17—C16	121.1 (2)
C1—O1—Na1	123.52 (14)	C18—C17—H17	119.4
C7—O2—Cu1	125.64 (11)	C16—C17—H17	119.4
C7—O2—Na1	128.90 (11)	C17—C18—C19	120.3 (2)
Cu1—O2—Na1	103.06 (5)	C17—C18—H18	119.9
C21—O3—Cu1	126.40 (12)	C19—C18—H18	119.9
C21—O3—Na1	127.68 (12)	C20—C19—C18	120.3 (2)
Cu1—O3—Na1	105.50 (6)	C20—C19—H19	119.8
C20—O4—C22	118.06 (18)	C18—C19—H19	119.8
C20—O4—Na1	121.02 (11)	O4—C20—C19	126.06 (19)
C22—O4—Na1	120.19 (15)	O4—C20—C21	112.95 (16)
C23—O5—Na1	120.05 (18)	C19—C20—C21	121.0 (2)
C23—O5—H5A	107 (3)	O3—C21—C16	125.32 (17)
Na1—O5—H5A	133 (3)	O3—C21—C20	116.79 (17)
O1—C1—H1A	109.5	C16—C21—C20	117.89 (17)
O1—C1—H1B	109.5	O4—C22—H22A	109.5
H1A—C1—H1B	109.5	O4—C22—H22B	109.5
O1—C1—H1C	109.5	H22A—C22—H22B	109.5
H1A—C1—H1C	109.5	O4—C22—H22C	109.5
H1B—C1—H1C	109.5	H22A—C22—H22C	109.5
O1—C2—C3	125.50 (17)	H22B—C22—H22C	109.5
O1—C2—C7	112.95 (15)	O5—C23—H23A	109.5
C3—C2—C7	121.55 (17)	O5—C23—H23B	109.5
C2—C3—C4	120.32 (18)	H23A—C23—H23B	109.5
C2—C3—H3	119.8	O5—C23—H23C	109.5
C4—C3—H3	119.8	H23A—C23—H23C	109.5
C5—C4—C3	120.15 (17)	H23B—C23—H23C	109.5

### Hydrogen-bond geometry (Å, °)

**Table:**

*D*—H···*A*	*D*—H	H···*A*	*D*···*A*	*D*—H···*A*
O5—H5A···Cl1^i^	0.86 (2)	2.34 (2)	3.193 (2)	172 (4)
C10—H10···Cl1^ii^	0.93	2.82	3.729 (2)	165

Symmetry codes: (i) −*x*+1/2, *y*−1/2, −*z*+3/2; (ii) −*x*+1, −*y*, −*z*+1.
